# Impact of and Reasons for Not Performing Exercise Training After an Acute Coronary Syndrome in the Setting of an Interdisciplinary Cardiac Rehabilitation Program: Results From a Risk-Op- Acute Coronary Syndrome Ambispective Registry

**DOI:** 10.3389/fphys.2021.768199

**Published:** 2021-11-24

**Authors:** Ignacio Cabrera-Aguilera, Consolació Ivern, Neus Badosa, Ester Marco, Luís Salas-Medina, Diana Mojón, Miren Vicente, Marc Llagostera, Nuria Farré, Sonia Ruiz-Bustillo

**Affiliations:** ^1^Facultat de Medicina i Ciències de la Salut, Unitat de Biofísica i Bioenginyeria, Universitat de Barcelona, Barcelona, Spain; ^2^Heart Diseases Biomedical Research Group, IMIM (Hospital del Mar Medical Research Institute), Barcelona, Spain; ^3^Department of Human Movement Sciences, Faculty of Health Sciences, School of Kinesiology, Universidad de Talca, Talca, Chile; ^4^Cardiac Rehabilitation Unit, Department of Cardiology, Hospital del Mar, Barcelona, Spain; ^5^Cardiac Rehabilitation Unit, Physical Medicine and Rehabilitation Department, Parc de Salut Mar (Hospital del Mar – Hospital de l’Esperança), Barcelona, Spain; ^6^Rehabilitation Research Group, Hospital del Mar Medical Research Institute, Barcelona, Spain; ^7^Department of Medicine, Universitat Autònoma de Barcelona, Barcelona, Spain; ^8^Department of Medicine, Universitat Pompeu Fabra, Barcelona, Spain

**Keywords:** acute coronary syndrome, cardiac rehabilitation, exercise training, rehabilitation adherence, ischemic heart disease, event-free survival

## Abstract

**Background and Aims:** Exercise training (ET) is a critical component of cardiac rehabilitation (CR), but it remains underused. The aim of this study was to compare clinical outcomes between patients who completed ET (A-T), those who accepted ET but did not complete it (A-NT), and those who did not accept to undergo it (R-NT), and to analyze reasons for rejecting or not completing ET.

**Methods and Results:** A unicenter ambispective observational registry study of 497 patients with acute coronary syndrome (ACS) was carried out in Barcelona, Spain, from 2016 to 2019. The primary endpoint was a composite of all-cause mortality, hospitalization for ACS, or need for revascularization during follow-up. Multivariable analysis was carried out to identify variables independently associated with the primary outcome. Initially, 70% of patients accepted participating in the ET, but only 50.5% completed it. The A-T group were younger and had fewer comorbidities. Baseline characteristics in A-NT and R-NT groups were very similar. The main reason for not undergoing or completing ET was rejection (reason unknown) or work/schedule incompatibility. The median follow-up period was 31 months. Both the composite primary endpoint and mortality were significantly lower in the A-T group compared to the A-NT and R-NT (primary endpoint: 3.6% vs. 23.2% vs. 20.4%, *p* < 0.001, respectively; mortality: 0.8% vs. 9.1% vs. 8.2%, *p* < 0.001; respectively). During multivariable analysis, the only variables that remained statistically significant with the composite endpoint were ET completion, previous ACS, and anemia.

**Conclusion:** Completion of ET after ACS was associated with improved prognosis. Only half of the patients completed the ET program, with the leading reasons for not completing it being refusal (reason unknown) and work/schedule incompatibility. These results highlight the need to focus on the needs of patients in order to guarantee that structural barriers to ET no longer exist.

## Introduction

According to the World Health Organization, ischemic heart disease was the top cause of death in 2000 and 2019, responsible for 16% of total deaths each year. Moreover, fatalities due to the disease have seen the most significant increase, rising from over 2 million in 2000 to nearly 9 million in 2019 (Nowbar et al., 2019; World Health Organization [WHO], 2021). Despite the improvements in primary prevention, diagnosis, and treatments (Widimsky et al., 2019), the long-term prognosis of ischemic heart disease remains poor, with a high rate of acute coronary syndrome (ACS), need for coronary revascularization, and death (Collet et al., 2021). Cardiac rehabilitation (CR) is an integral and complex intervention that comprises exercise training (ET), behavioral change, psychological support, and other strategies to control traditional risk factors of cardiovascular disease. Several studies and meta-analyses have shown that CR is associated with better prognosis. Therefore, CR has a class I recommendation from the European Society of Cardiology for the management of a cardiac ischemic event (Anderson et al., 2016; Ibanez et al., 2018; Ji et al., 2019; Collet et al., 2021). Unfortunately, CR remains underused due to multiple reasons (Bethell et al., 2008; Balady et al., 2011; Serón et al., 2019). On the one hand, many countries and regions do not have CR programs. On the other hand, patients are often unwilling or unable to enroll in CR, especially the ET component. Indeed, only 50% of patients referred for CR end up participating in ET (Dunlay et al., 2014). Although several studies have analyzed the difference between patients who accept to undergo ET and those who do not, few studies have studied patients who begin ET but do not complete it. Thus, it is unknown whether patients who begin ET but do not complete it have different baseline characteristics and outcomes than patients who complete ET and those who outrightly reject it. Patient-centered care (PCC) has been proposed as a central component for a sustainable, affordable, and high-quality healthcare approach. It underlines the importance of understanding the patient’s capabilities and resources in order to engage the patient to participate in care (Ekman et al., 2011). PCC efficiency has been demonstrated in several conditions and care levels, including ACS (Fors et al., 2016; Wolf et al., 2019). To provide optimal PCC, it is critical to know why patients choose not to participate in CR. This knowledge would improve prognosis.

This study aimed to assess whether there were epidemiological differences between patients referred for CR according to exercise compliance and initial attitude toward ET, analyze the reasons for rejecting or not completing ET, and explore the prognostic impact of each group.

## Materials and Methods

### Study Design and Population

A total of 497 patients from an ambispective observational registry study carried out at the Hospital del Mar, Barcelona, Spain from November 2016 to September 2019 were included in this analysis. Patients with ST-elevation acute myocardial infarction (STEMI), non-ST-elevation acute myocardial infarction (non-STEMI), and unstable angina (UA) were included. The diagnosis was made according to the European Heart Association guidelines (Roffi et al., 2015; Ibanez et al., 2018). All patients from our health area were invited to participate in the CR program (CRP). The only exclusion criteria were being from other health areas and the existence of a severe language barrier.

### Cardiac Rehabilitation Program

The CRP at the Hospital del Mar is an interdisciplinary program that combines interventions performed by cardiologists, nurses, rehabilitation physicians, physiotherapists, and psychiatrists. All patients with ACS receive education on healthy habits during the initial ACS hospitalization, or shortly after discharge for those hospitalized in other hospitals. Specialized nurses undertake follow-up visits 3 and 12 months after inclusion in the CRP. Patients attend weekly group sessions with healthcare professionals aimed at reinforcing their health education, with a particular focus on understanding the pathophysiology of ACS, the role of cardiovascular risk factors, and the importance of optimal risk factor management, mainly through physical activity, control of anxiety, smoking cessation, and adherence to guideline-recommended drugs.

All patients are referred to participate in ET. Patient functional status is assessed on enrollment by a treadmill test. Rehabilitation physicians prescribe the levels and types of exercise for patients according to their characteristics and risk stratification. The ET intervention consists of 25 1-h sessions—five times per week (Monday–Friday from 9 to 10 a.m.) for 5 weeks. Each session starts with a 5-min warm-up period followed by 30 min of cycling (80% of maximal heart rate achieved in an exercise test), 20 min of resistance muscle training of upper and lower limbs (weight at 10 RM), and a 5-min cool-down period. An expert physiotherapist supervises sessions with continuous heart rate and pulse oximetry monitoring. The workload progression is adjusted weekly according to the patient’s tolerance (Borg perceived effort scale). Dropout in ET is defined as attending no more than 50% of sessions (Worcester et al., 2004).

### Study Variables

We collected baseline demographic and clinical data and follow-up events, viz.: age, gender, BMI, risk factors and comorbidities, diagnostics, and basal hospitalization data. Patients were classified into three groups: those who completed the ET (A-T), those who outrightly rejected ET (R-NT), and those who accepted but did not complete ET (A-NT). We collected the reasons for not wanting to perform the ET. When the cause was not established in the medical record, it was classified as “rejected-reason unknown.” The primary endpoint was a frequently used composite of all-cause mortality, hospitalization due to ACS, or need for new revascularization during follow-up (Bainey et al., 2020; Turgeon et al., 2020).

### Statistics

Continuous variables were expressed as mean ± standard deviation (SD) or median and interquartile range (IQR) based on normality distribution assessed by the Kolmogorov–Smirnov test. Categorical variables were expressed as percentages. Differences in baseline characteristics between groups previously defined by level participation in CRP were tested using the χ^2^-test (categorical variables); and Student’s *t*-test or the Mann–Whitney *U*-test, one-way analysis of variance or the Kruskal–Wallis test for continuous variables. Univariable and multivariable analyses were performed using the Cox proportional hazard regression model to examine the association between ET participation and outcomes. Variables with an overall significance value of *p* < 0.05 were used for multiple Cox regression analysis to identify the strongest predictors of event-free survival. The model was adjusted for potential confounders selected by stepwise forward inclusion among patient characteristics previously defined. The variables included in the multivariable analysis have also been associated with the endpoint in several studies (Arnold et al., 2015; Bainey et al., 2020; Turgeon et al., 2020). The log-rank test compared the Kaplan–Meier survival curves. All analysis was performed using IBM SPSS Statistics v25 (Armonk, NY, United States) and GraphPad Prism 8.0 (San Diego, CA, United States). For all tests, *p* < 0.05 was considered as statically significant.

### Ethics

The study was designed in compliance with the ethical principles set forth in the Declaration of Helsinki. The Ethics Committee of the Hospital del Mar (Parc de Salut Mar) approved the study (N° 2018/7896/I). The data included in this study incorporated data from a registry collection carried out prospectively between July 2018 and September 2019, with all the patients providing written informed consent. Retrospective data collection was carried out between November 2016 and June 2018, with the Ethical Committee approving the inclusion of these group of patients to increase the sample size but waiving the need for written informed consent by this group, given that the same protocol had been carried out before.

## Results

### Patients’ Baseline Clinical Characteristics

**FIGURE 1:**
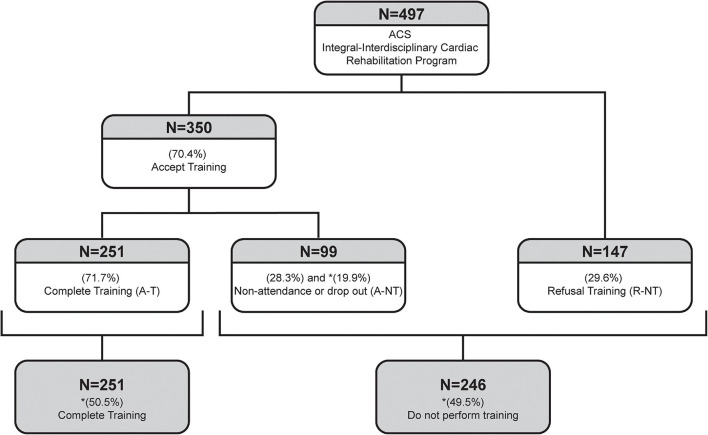
Flow chart of patients in the cardiac rehabilitation program. ACS, Acute coronary syndrome; R-NT, Rejected and did not perform training; A-T, Accepted and did exercise training; A-NT Accepted but did not do/complete training; CV, Cardiovascular. Data are expressed in percentages to the preceding category. Data with an asterisk (*) were calculated to refer to the whole cohort.

The flow chart of the 497 patients included in this study is depicted in Figure 1. Although 70% of patients initially accepted to participate in the ET, 28% of this group did not complete the ET for various reasons. Therefore, only 50.5% of the whole population completed the ET.

Baseline clinical characteristics of the study population are summarized in Table 1. In brief, most of the patients were men with a diagnosis of STEMI, 1-vessel disease, and preserved ejection fraction. No significant differences were found when comparing both sexes. Patients who completed ET (A-T) were younger and had fewer comorbidities. Patients in R-NT and A-NT groups had similar baseline characteristics.

**TABLE 1:** Baseline characteristics of patients in the cardiac rehabilitation program and in the three groups.

Characteristic/Variable	Overall (*n* = 497)	A-T (*n* = 251)	A-NT (*n* = 99)	R-NT (*n* = 147)	*p-*value*	*p-*value**	*p-*value***	*p-*value****
**Anthropometric**								
Age (years)	62.6 ± 11.9	60.9 ± 10.7	62.5 ± 11.2	65.6 ± 13.6	0.001	0.203	0.056	<0.001
Women	93 (18.7)	45 (17.9)	16 (16.2)	32 (21.8)	0.490	0.695	0.276	0.349
BMI (Kg/m^2^)	27.1 (24.7–30.1)	27.0 (24.7–29.4)	27.3 (23.9–30.9)	27.0 (24.6–30.4)	0.781	0.523	0.901	0.618
**Risk factors and comorbidities**								
Hypertension	281 (56.5)	126 (50.2)	58 (58.6)	97 (66.0)	0.008	0.157	0.238	0.002
Hyperlipidemia	324 (65.2)	168 (66.9)	59 (59.6)	97 (66.0)	0.419	0.195	0.308	0.847
Diabetes mellitus	143 (28.8)	55 (21.9)	32 (32.3)	56 (38.1)	0.002	0.042	0.354	0.001
Current smoker	205 (41.3)	95 (37.9)	46 (46.5)	64 (43.5)				
Previous smoker > 1 year	146 (29.4)	82 (32.7)	25 (25.3)	39 (26.5)	0.705	0.406	0.953	0.585
Previous smoker < 1 year	20 (4.0)	11 (4.4)	3 (3.0)	6 (4.1)				
COPD	40 (8.1)	15 (6.0)	10 (10.0)	15 (10.2)	0.230	0.177	0.979	0.123
Cerebrovascular disease	23 (4.6)	9 (3.6)	5 (5.1)	9 (6.1)	0.496	0.529	0.722	0.240
Peripheral vascular disease	39 (7.9)	14 (5.6)	3 (3.0)	22 (15.0)	<0.001	0.318	0.002	0.002
Anemia	87 (17.5)	29 (11.6)	20 (20.2)	38 (25.9)	0.001	0.036	0.306	<0.001
Renal disease	29 (5.8)	8 (3.2)	5 (5.1)	16 (10.9)	0.006	0.406	0.108	0.002
Arthropathy	10 (2.0)	2 (0.8)	2 (2.0)	6 (4.1)	0.079	0.332	0.371	0.024
**Diagnostics and basal hospitalization**								
STEMI	220 (44.3)	120 (47.8)	41 (41.4)	59 (40.1)				
NSTEMI	174 (35.0)	93 (37.1)	38 (38.4)	43 (29.3)	0.007	0.414	0.139	0.001
Unstable angina	103 (20.7)	38 (15.1)	20 (20.2)	45 (30.6)				
Hospitalization length (days)	5 (4–7)	5 (3–6)	5 (4–7)	5 (4–7)	0.116	0.169	0.816	0.055
Previous ACS-MI	99 (19.9)	34 (13.6)	21 (21.2)	44 (29.9)	<0.001	0.076	0.128	<0.001
One vessel disease	263 (52.9)	144 (57.4)	42 (42.4)	77 (52.4)				
Two vessel disease	126 (25.4)	59 (23.5)	34 (34.3)	33 (22.5)	0.192	0.138	0.200	0.474
Three vessel disease	87 (17.5)	40 (15.9)	20 (20.2)	27 (18.4)				
Ejection fraction (%)	58 (52–62)	58 (51–62)	60 (52–63)	59 (54–62)	0.451	0.545	0.695	0.210

Data are mean ± SD, median (IQR), or n (%). p* values for comparing all three groups (null hypothesis: all three groups had the same characteristics). p** values only apply to the comparison of Accepted and did exercise training (A-T) vs. Accepted but did not do/complete training (A-NT) groups. p*** values only apply to the comparison of A-NT vs. Rejected and did not perform training (R-NT) groups. p**** values only apply to the comparison of A-T vs. R-NT groups.

*BMI, Body mass index; COPD, chronic obstructive pulmonary disease; CV, Cardiovascular; STEMI, ST-elevation myocardial infarction; NSTEMI, non-ST-elevation myocardial infarction; ACS-MI, Acute coronary syndrome-myocardial infarction.*

### Reasons for Not Completing the Exercise Training Component of the CR Program

The main reason for outrightly rejecting to participate in or not completing the ET was rejection-reason unknown. Table 2 presents the reasons for non-participation in and non-completion of the ET component of the CRP. Reasons for not completing the ET were qualitatively somewhat different in women and men.

**TABLE 2:** Description of reasons by sexes for not doing the exercise training in the group that initially refused and those who initially accepted but did not complete exercise training.

R-NT (*n* = 147)	Female (*n* = 32)	Male (*n* = 115)	All	A-NT (*n* = 99)	Female (*n* = 16)	Male (*n* = 83)	All
Rejection-reason unknown	12 (37.5)	45 (39.1)	57 (38.8)	Rejection-reason unknown	6 (37.5)	38 (45.8)	44 (44.4)
Functional problems	6 (18.8)	16 (13.9)	22 (15.0)	Medical recommendation	8 (50.0)	21 (25.3)	29 (29.3)
Job/work incompatibility	7 (21.9)	14 (12.2)	21 (14.3)	Job/work incompatibility	2 (12.5)	17 (20.5)	19 (19.2)
Schedule and distance	1 (3.1)	20 (17.8)	21 (14.3)	CV disease	0 (0.0)	4 (4.8)	4 (4.0)
Medical recommendation	4 (12.5)	8 (7.0)	12 (8.2)	Schedule and distance	0 (0.0)	2 (2.4)	2 (2.0)
Language barrier	0 (0.0)	9 (7.8)	9 (6.1)	SARS-CoV-2 contingency	0 (0.0)	1 (1.2)	1 (1.0)
Social problems	2 (6.3)	3 (2.6)	5 (3.4)				

*Data are n (%). CV, Cardiovascular.*

Only four patients (4% of A-NT) could not complete the ET due to cardiovascular complications: one patient with atrial fibrillation, one with a high-risk treadmill test, one due to chest pain, and one with ACS. These events did not occur during the ET. Work incompatibility and distance/schedule were also relevant reasons for not performing ET in both the A-NT and R-NT groups. It is noteworthy that none of the A-T patients experienced a CV complication while exercising, confirming that ET was safe in this group of patients.

### Prognosis

During a median follow-up of 31 (23–39) months, 12% of all patients showed a composite outcome of all-cause death, new ACS, or need for coronary revascularization. The A-T group showed an excellent prognosis. In this group, the mortality rate was < 1%. The prognoses in the A-NT and R-NT groups were similar and worse than that in the A-T group (Table 3).

**TABLE 3:** Outcomes of patients included in the cardiac rehabilitation program and in the three groups.

Outcome	Overall (*n* = 497)	A-T (*n* = 251)	A-NT (*n* = 99)	R-NT (*n* = 147)	*p-*value*	*p-*value**	*p-*value***	*p-*value****
Composite Outcome	62 (12.5)	9 (3.6)	23 (23.2)	30 (20.4)	<0.001	<0.001	0.597	<0.001
Death	23 (4.6)	2 (0.8)	9 (9.1)	12 (8.2)	<0.001	<0.001	0.798	<0.001
New ACS	25 (5.0)	5 (2.0)	7 (7.1)	13 (8.8)	0.006	0.019	0.618	0.002
Revascularization	36 (7.2)	8 (3.2)	12 (12.1)	16 (10.9)	0.002	0.001	0.765	0.002
All-cause hospitalization	168 (33.8)	61 (24.3)	48 (48.5)	59 (40.1)	<0.001	<0.001	0.195	0.001
All-CV hospitalization	81 (16.3)	22 (8.8)	19 (19.2)	40 (27.2)	<0.001	0.006	0.149	<0.001

ACS, Acute coronary syndrome; CV, Cardiovascular. Data are n (%). p* values for comparing all three groups (null hypothesis: all three groups had the same characteristics). p** values only apply to the comparison of Accepted and did exercise training (A-T) vs. Accepted but did not do/complete training (A-NT) groups. p*** values only apply to the comparison of A-NT vs. Rejected and did not perform training (R-NT) groups. p**** values only apply to the comparison of A-T vs. R-NT groups.

After adjustment for age, sex, anemia, hypertension, diabetes mellitus, renal disease, previous ACS, peripheral vascular disease, type of ACS, and ET compliance, the only variables that remained statistically significant with the composite endpoint on multivariable Cox regression analysis were ET compliance [A-NT, HR 5.6 (95% CI 2.75–13.05); R-NT, HR 4.32 (2.00–9.29)], previous ACS and anemia (Table 4).

**TABLE 4:** Univariable and multivariable Cox regression analyses for the composite endpoint.

	Univariable HR (95% CI)	*p*-value	Adjusted HR (95% CI)	*p*-value
A-T	1 (ref)		1 (ref)	
A-NT	7.12 (3.30–15.40)	<0.001	5.60 (2.75–13.05)	<0.001
R-NT	5.88 (2.79–12.38)	<0.001	4.32 (2.00–9.29)	<0.001
Previous ACS-MI	2.79 (1.68–4.63)	<0.001	1.81 (1.07–3.08)	0.028
Anemia	3.48 (2.09–5.78)	<0.001	2.33 (1.37–3.97)	0.002
Renal disease	2.96 (1.46–6.01)	0.003		
Age (years)	1.04 (1.02–1.06)	0.001		
Hypertension	1.97 (1.14–3.42)	0.015		
Diabetes mellitus	2.02 (1.23–3.34)	0.006		
Female	1 (ref)			
Male	1.22 (0.66–2.25)	0.521		
Peripheral vascular disease	1.33 (0.57–3.10)	0.504		
STEMI	1 (ref)			
NSTEMI	1.43 (0.79–2.58)	0.241		
Unstable angina	1.88 (1.00–3.53)	0.050		

*The composite endpoint includes all-cause mortality, hospitalization due to ACS, or the need for new revascularization during follow-up. A-T, Accepted and did exercise training; A-NT, Accepted but did not do/complete training; R-NT, Rejected and did not perform training (R-NT) groups; STEMI, ST-elevation myocardial infarction; NSTEMI, non-ST-elevation myocardial infarction; ACS-MI, Acute coronary syndrome-myocardial infarction.*

**FIGURE 2:**
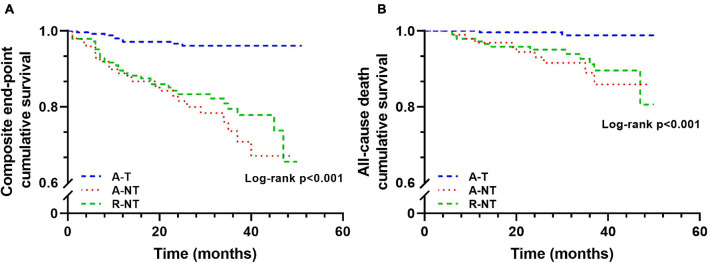
Kaplan–Meier curves for long-term outcome grouped by ET groups (**A**, composite endpoint cumulative survival; **B**, cumulative survival). A-T, Accepted and did exercise training; A-NT, Accepted but did not do/complete training; R-NT, Rejected and did not perform training. The composite endpoint includes all-cause mortality, hospitalization due to ACS, or the need for new revascularization during follow-up.

Figure 2 shows the Kaplan–Meier survival curves for the composite endpoint and all-cause mortality in the three groups; the A-T group shows better survival in both curves.

## Discussion

Although 70% of patients accepted to participate in ET after ACS during a CRP, only 50.5% of the total patients completed the ET protocol. Patients who completed ET (A-T) were younger and had fewer comorbidities. Their prognosis was excellent, with mortality < 1% at a median follow-up of 31 months. Patients who did not complete ET (A-NT and R-NT) had similar baseline characteristics and prognosis, which was worse than that of the A-T group. The main reason for not participating in ET was refusal from the patients and work and schedule/distance incompatibilities, highlighting the need to find ways to engage patients in ET and increase ET logistic capabilities.

Although the initial number of patients who accepted ET was 70%, only 50.5% completed the ET protocol, a rate similar to the 52.5% participation obtained in a population-based surveillance study of patients after their first ever myocardial infarction (Dunlay et al., 2014). Patients who completed the ET were younger and with fewer comorbidities, observations that are consistent with those of other studies (Dunlay et al., 2014; Pardaens et al., 2017). This finding might seem counterintuitive because higher-risk patients (i.e., those with comorbidities) should be more prone to accept ET to decrease their risk (Bainey et al., 2020). Several reasons might explain why this was not the case in this study. On the one hand, patients who completed ET (A-T) might be fitter due to their being younger and having fewer comorbidities, and more open to going to the gym to exercise. Moreover, this group had less history of ACS and presented more frequently with STEMI. This acute presentation might have prompted them to accept any option that would improve their prognosis. On the other hand, the A-NT and R-NT patients were older and had more comorbidities, some of which might have hindered their ability to exercise (e.g., peripheral vascular disease and anemia), and even though the exercise was adapted to the needs of the patients, some patients might not have found it valuable and thus rejected it. Surprisingly, there is not much information in the literature on patients who initially accept to undergo ET but do not start or finish it. Given that they accounted for 28% of the patients who initially accepted to undergo ET in this study, it is a group that should not be overlooked. Their baseline characteristics were similar to those of the R-NT group except for a lower prevalence of peripheral vascular disease.

Reasons for low rates of use of CR are very diverse among different countries and regions. Barriers to participation in ET are classified as inherent to the patient’s characteristics or related to accessibility of the CRP system (Ruano-Ravina et al., 2016). For instance, gender, age, comorbidities, disease perception, social class, education level or accessibility, and proximity to the CRP center plays a crucial role in adherence to ET. For women, barriers to CR participation are multiple and complex, explaining their low participation rates (Forsyth and Deaton, 2020; Vidal-Almela et al., 2020). While our study included 18.71% of female, some studies reported an 11–20% lower enrollment, and women were more likely to withdraw from a program than men (35% vs. 29%) (Marzolini et al., 2008; Balady et al., 2011). Reasons for not accepting to undergo ET, or for accepting but not completing it, were quite similar in the R-NT and A-NT groups. The main reason in both groups (41% of patients) was the unwillingness of the patients to participate, with no explanations given for this unwillingness. This finding is consistent with those of other studies where 47.5% of patients did not participate in ET (Worcester et al., 2004; Dunlay et al., 2014). The other reasons more frequently mentioned were job/work incompatibility and schedule and distance, which accounted for 26% of patients that did not participate or withdrew from ET. Reasons for not completing ET were different between men and women. They also varied according to the time of assessment (initial refusal vs. once accepted but not completed), emphasizing the need to tailor ET to the needs of patients. Only 27% of patients were excluded from ET due to medical reasons, such as the presence of comorbidities. This percentage is significant and highlights the need to focus on what can be changed in the system to guarantee that structural barriers to ET disappear, so that genuine PCC can be carried, which would greatly increase the number of patients who complete ET and also bridge the gender gap in participation. PCC can help identify intangible barriers, such as those reported by Resurrección et al. (2017), in which traditional ways of CRP with predominantly male presence could make women feel uncomfortable. Finally, our results showed an excellent long-term prognosis in patients who completed ET, with a composite of all-cause mortality, hospitalization due to ACS, or need for new revascularization in only 3.6%, after more than 2 years of follow-up, and a mortality of 0.8%. In contrast, patients in the R-NT and A-NT groups showed at least four times more risk with the composite endpoint and all-cause mortality, with no significant difference between the two groups. These results are consistent with those of other studies that found that the risk of death or having a recurrent CV event in patients that do not complete ET is over double the risk in patients who complete it (Pardaens et al., 2017).

## Limitations

As a single-center study, the results might not apply to other settings. Some of the patients were included retrospectively, and that might have potentially led to bias. Still, given that all patients followed the same protocol and that the information was documented in the medical record in a structured way, we believe that the risk of bias in this study is negligible. The lack of ET schedule flexibility in our center might have affected adherence. Patients’ participation might have been higher if we had more liberty to schedule their ET time. Due to the low number of women in our sample size, we cannot comment on the gender differences in ET participation.

## Conclusion

Completion of ET after ACS is associated with an improved prognosis. Only half of the patients in this registry study completed the ET component in the setting of an interdisciplinary CRP, although 70% of patients accepted participating in ET. Patients who completed ET (A-T) were younger and had fewer comorbidities. Their prognosis was excellent, with mortality < 1% at a median follow-up of 31 months. Patients who outrightly rejected participating in ET and those who began but did not complete it had similar baseline characteristics and prognosis, which was worse than that of the A-T group. The main reasons for not participating in AT in both groups were refusal-reason unknown and work and schedule/distance incompatibility. These results highlight the need to focus on the needs of the patients in order to minimize structural barriers to ET.

## Data Availability Statement

The original contributions presented in the study are included in the article/supplementary material, further inquiries can be directed to the corresponding author/s.

## Ethics Statement

The study was designed in compliance with the ethical principles set forth by the Declaration of Helsinki. The Ethics Committee of the Hospital del Mar (Parc de Salut Mar) approved the study (N° 2018/7896/I). The patients/participants provided their written informed consent to participate in this study.

## Author Contributions

SR-B and NB contributed to the conception or design of the work. NB, CI, MV, ML, DM, and IC-A contributed to the data acquisition. NF and IC-A performed data analysis and drafted the manuscript. NF, SR-B, and IC-A contributed to data interpretation. EM, SR-B, and LS-M critically revised the manuscript. All authors approved the final version of the manuscript and are accountable for all aspects of the work.

## Conflict of Interest

The authors declare that the research was conducted in the absence of any commercial or financial relationships that could be construed as a potential conflict of interest.

## Publisher’s Note

All claims expressed in this article are solely those of the authors and do not necessarily represent those of their affiliated organizations, or those of the publisher, the editors and the reviewers. Any product that may be evaluated in this article, or claim that may be made by its manufacturer, is not guaranteed or endorsed by the publisher.
